# Individual vs. Team Sports—What’s the Better Strategy for Meeting PA Guidelines in Children?

**DOI:** 10.3390/ijerph182212074

**Published:** 2021-11-17

**Authors:** Michal Kudlacek

**Affiliations:** Faculty of Physical Culture, Palacký University Olomouc, 77111 Olomouc, Czech Republic; michal.kudlacek@upol.cz; Tel.: +420-585-636-254

**Keywords:** lifestyle, education, health, leisure, programming

## Abstract

There is insufficient evidence from previous studies dealing with structure of sport preferences referring to the interconnection between individual factors (socio-economic status, organized/structured physical activity (PA), location, etc.), although these factors can considerably influence total level of PA as well as the structure of sport preferences. The study investigated associations between PA frequency and specific sports activities according to the intensity with the impact on leisure, sport, and education domain, using data from an international health behavior in school-aged children survey. Participants were fifth and ninth grade students in the Czech Republic (seven schools) and Slovakia (nine schools). The results showed a significant association between intensity in team sports and PA frequency per week. Those who participated in high-intensity team sports were 2.5 times more likely to be more physically active.

## 1. Introduction

The health benefits of a physically active lifestyle have been strongly emphasized [1], and sports participation in childhood and adolescence [2,3] has been reported to increase the probability of a high level of physical activity (PA) later in life [4,5,6,7,8,9]. Previously conducted surveys on sports preferences (i.e., sport activities people prefer to engage in) found visible dynamics in the structural development of sports preferences [10,11,12,13,14,15].

There is insufficient evidence from previous studies that examined the structure of sports preferences and the associations between individual factors (e.g., socioeconomic status, organized or structured PA, location), although these factors can considerably influence total PA levels and sports preference structure. However, there is no single factor that would explain the heterogeneity of the structure of sports preferences in a specific population in particular. Acquired data can provide an approximate prognosis of demanded activities on the national, regional, and local levels. These data can also serve as a tool to audit the usage of existing available sources of PA, facilities and/or programs, which can form the basis for developing new facilities or innovating existing ones.

Matching available sports opportunities with the PA of a particular population is a likely predictor of whether people will regularly participate in sports. Surveys on the sport preferences should be conducted by considering a number of dimensions for sports participation and PA. One of the key dimensions is the intensity of an activity. Activity intensity is a crucial factor in an individual’s decision-making process, as some people prefer vigorous intensity, some prefer moderate intensity, and some do not distinguish between these types and prefer the activity on its own [16,17,18,19]. The mainstream gender view on this issue indicates that men prefer more intensive activities than women. Women and girls show higher preferences for moderate activities, and this generally increases with age. Moderate activities are also preferred by individuals with lower PA levels, and those who do not participate in organized sports. Contrastingly, men and boys tend to preferred vigorous PA, although this generally decreases as age increases [20,21,22,23,24].

Another key dimension is the competitiveness of the activity, which is also a crucial influencing factor for participation in a particular activity. Men and boys tend to show a preference toward exclusively participating in more competitive activities as compared to women and girls. This preference generally decreases with age and is more common among those with less than a high school education. In contrast, non-competitive activities are generally preferred by women and girls, and those who do not participate in organized sports [25,26,27].

Activity type is another relevant dimension, as it is important whether an activity is performed individually or in a group. This dimension cannot be divided according to gender; however, there are other factors within this dimension that can influence individual preferences. Team activities are generally preferred by individuals with less education and lower income, while individual activities are usually preferred by individuals with higher socio-economic status and a higher level of education [11,13,28,29,30].

The sports preferences area (as a part of personality psychology) is closely associated with other areas, such as urban planning, policy-making, facility management, PA program planning, physical education (PE) curriculum planning, intervention programming, health promotion, and active lifestyle implementation among all populations, not only children and adolescents. This area should be seen as a sensitive one because of particular factors in each population group, such as age, gender, socioeconomic status, education level, residence type, family size, family influence, peer influence, family affluence status, nutritional habits and cultural aspects. Notably, there is no individual more or less important factor influencing this complex phenomenon of sports preferences. The power of each sector should be unified into the same effort with a shared aim, which is the implementation of PA preferences into the lifestyle of all individuals as lifetime PA. This kind of approach should serve as a “tool” for not only meeting PA recommendations but also for providing sufficient fulfillment of the mental, social, and spiritual dimension of health.

Researchers in the PE field have indicated that identifying and understanding activity participation are critical to the promotion of both current and lifelong PA participation [31,32,33,34]. Among many factors, children’s attitudes are considered to be a key influence on PA participation [31,32,35,36,37]. Research has indicated that children who have more positive attitudes toward PA are more likely to participate in PA outside of school [31,32,38,39,40,41], and demonstrate higher PA levels overall [35,42], as compared with children who have less positive attitudes.

The aim of this study was to investigate and analyze associations between amount of weekly PA and specific types of sports activities as assessed by intensity.

## 2. Methods

### 2.1. Participants and Settings

This study was based on the International Health Behavior in School-aged Children (HBSC) study and was consistent with its methodology (hbsc.org). It was conducted in the Czech Republic and Slovakia. This study was preceded by a pilot study which included the administration of questionnaires and focus groups in both countries. Based on data obtained in the pilot study, the final set of questions was compiled. We contacted 16 primary schools located in rural and urban areas in the Olomouc region, Czech Republic (seven schools, including two from rural area) and the Kosice region, Slovakia (nine schools; including three from rural areas). The schools were randomly chosen to create a representative sample. We reached a response rate of 100% on the school level, as all the contacted schools agreed to participate. Questionnaires were administrated to students in the fifth and ninth grades by trained research assistants during regular class time without the teachers present.

We obtained data from 867 adolescents (age range 11 to 15 years) in the Czech Republic (response rate: 83.20%) and Slovakia (response rate: 74.14%). Non-responses were primarily due to illness or a lack of parental consent for participation. The final sample consisted of 426 Czech (49.5% boys) and 441 Slovak (55.6% boys) primary school students in the 5th and 9th grades.

### 2.2. Measurements

PA was measured using an item that asked adolescents about the number of days over the past week they had been physically active for a total of at least 60 min per day. The question was preceded by explanatory text that defined moderate-to-vigorous PA as “any activity that increases your heart rate and makes you become out of breath some of the time,” with examples offered such as running, inline skating, cycling, dancing, swimming, and ice skating [43]. Responses ranged from 0 to 7 days. Using a cut-off point of 5 days of PA per week, the responses were dichotomized as (1) active (six or seven days per week) and (2) less active (five days per week or less).

Detailed insight into the area of PA and its content were assessed through two categories: (1) individual sports and (2) team sports. Both categories contained a list of proposed activities and a free option for participants to provide their own answer. The list of proposed individual sports included athletics, badminton, bowling, skating, cycling, golf, swimming, sports gymnastics, squash, and tennis. The list of proposed team sports included basketball, floorball, football, ultimate frisbee, handball, ice hockey, and volleyball. Both lists were created based on previous studies and sports preferences structures within both participating countries [13,14,44]. Each item from both areas (individual sports and team sports) had the same four possible responses: (1) I am not doing this activity, (2) I am doing this activity 2−3 times per month, (3) once a week, and (4) at least twice a week.

The Compendium of Physical Activities [45,46] was used for PA stratification. The key parameter of this stratification was PA intensity. The Compendium provides a coding scheme that links a five-digit code, representing specific activities performed in various settings, with the respective metabolic equivalent (MET) intensity levels. The measurement unit used in the Compendium is a MET, defined as the ratio of active metabolic rate to a standard resting metabolic rate of 1.0 (4.184 kJ) × kg^−1^ × h^−1^; 1 MET is considered an individual’s resting metabolic rate obtained during quiet rest. Based on values for each activity listed on the questionnaire, we stratified recorded activities as either (1) low intensity activities or (2) high intensity activities, so we could compare the amount of PA with the PA level of the participants.

Table 1 provides an overview of the intensity levels for particular activities, with cut-off points based on the frequency with which participants reported performing those activities in both categories (i.e., individual sports, team sports). These activities were ranked by intensity level according to the Compendium of Physical Activities, an update of activity codes and MET intensities by Ainsworth and colleagues [45,46]. Subsequently, we performed a median-based dichotomization.

### 2.3. Data Analysis

Data were analyzed using IBM SPSS, version 22 SPSS Inc., IBM Corp. Armonk, NY, USA). First, participants’ socio-demographic characteristics were described. Logistic regression analysis was then used to explore the associations of the individual and team activities in different intensities as the independent variable, with PA as the dependent variable (odds ratios [OR] and 95% confidence intervals [CI]).

### 2.4. Ethics Committee Statement

The study was conducted according to the ethical requirements formulated by the Agreement on Human Rights and Biomedicine (40/2000 Z.z.), and under the principles of the Declaration of Helsinki and the legal and regulatory requirements which apply to both countries. The study in Slovakia was approved by the Ethics Committee of the Medical Faculty at P. J. Safarik University in Kosice. Parents were informed about the study via the school administration and could opt for their children not to participate if they preferred. Participation in the study was fully voluntary and anonymous with no explicit incentives provided for participation. The study in the Czech Republic was approved by the Ministry of Health and the National Institute of Public Health. Czech legislation did not require this study to be approved by an ethics committee, as students completed the questionnaire anonymously.

## 3. Results

From the overall distribution within the research sample, which reflects the factors of gender, age, and country of origin, the specifics presented in Table 2 are evident. Regarding gender, girls were more active in the Czech Republic (25.6%), while boys were more active in Slovakia (34.7%). As for grade level, in the Czech Republic, fifth-grade boys were more active among boys (15.6%), and fifth-grade girls (7.1%) were also more active. In Slovakia, ninth-grade boys were more active than fifth-grade boys (18.3%), while fifth-grade girls (12.7%) were more active than ninth-grade girls.

Table 3 shows detailed results related to PA frequency when considering the exact numbers of active days per week. These results indicated that 25.2% of Czech respondents were physically active, compared to 27.7% of the respondents in Slovakia. In the Czech Republic, the largest proportion of boys engaged in PA three days per week (18.5%), while among boys in Slovakia, it was seven days per (21.6%). The largest proportion of girls in the Czech Republic were active seven days per week (19.1%), while among Slovakian girls, it was three days per week (19.9%). The overall distribution of physically active days per week was spread equally. The results also indicted that boys were less sedentary than girls, with higher percentages of girls than boys who were physically active only 0–2 days per week in both countries.

Table 4 shows descriptive statistics for the whole sample, as well as for each country separately. No significant differences were found in explored characteristics between participants in the Czech Republic and Slovakia. Accordingly, all other analyses were conducted using the whole sample.

Table 5 shows ORs and 95% CIs based on the results of logistic regression analysis. The results showed a significant association between intensity in team sports and number of days of PA per week. Involvement in high-intensity team sports increased the likelihood of being more physically active per week by 2.5 times. No significant association was found between intensity level and PA per week in individual sports.

The important and supportive evidence needed for complex overview represents the sports preferences survey. As shown in Table 6, there was a slight shift in the preferred activities of both boys and girls from age 11 to age 15, but the difference was not significant.

## 4. Discussion

The multidimensional analysis applied in the context of our research considered age, gender, PA type, and PA intensity. In evaluating PA, we have found some results that are in line with most previous studies, such as that younger people are more physically active compared to earlier years [21]. It can be stated that we are able to offer younger individuals’ activities that more closely match their preferences, while we are no longer as successful with older individuals. This finding conflicts with others, such as Dumith et al. [47] Undoubtedly, the age of the target group (i.e., adolescence) represents an important attribute; thus, we should make more intensive efforts to adequately monitor the preference for PA and sports of particular age groups.

Gender differences of PA participation were not found to be significant in our research, although this differed slightly between participating countries. In the Czech Republic, 25.4% of the participants were physically active, while in Slovakia, it was 27.4%. Further, in the Czech Republic, girls were more active (25.6%); however, in Slovakia it was boys (34.7%).

Over the last decade, studies were carried out that dealt with the research of sports preferences only marginally or very general [48,49,50,51]. Shields et al. [34], pointed out that barriers to PA participation have been studied more comprehensively than facilitators. Children’s preferences, a lack of knowledge and skills, parental behavior, inadequate facilities, lack of programs, staff capacity, and costs represents such important facilitators. Despite appeals from numerous researchers in the PE and sports fields, who have pointed to the need for continuous identification and monitoring of PA and a greater degree of understanding regarding the individual variables that affect PA participation, the state (amount, as well as quality) of the research in this area is still significantly insufficient.

Research on sports preferences in the context of frequency of PA per week has shown the significance of obtaining detailed insights into types of sports and physical activities. The typology of PA can be compared with marketing research, which addresses the current demand for specific products or fashion trends in various sectors [52,53,54,55]. Without this type of research (“marketing research” focused on the attribute of sport preferences in the context of PA), business strategies could not be implemented. However, in PA research, support, and intervention programs, the importance and the involvement of this important attribute seems to be rather minor.

Regarding preferred activities, football, floorball, and dodgeball/basketball, respectively, ranked highest among boys in both the fifth and ninth grades. These results were similar for girls in the fifth grade, while for girls in the ninth grade, volleyball came to the forefront of the preference rankings. This was in line with the findings of Asztalos et al. [56] and Bergier, Jerbier, and Wojtyla [57]. With respect to age, the favorite sports activities of adults are significantly different from those of children [58], which is mainly due to the involvement of lifelong activities in adults, instead of the competitive nature of the activities in adolescents.

The key factor in our research design was PA intensity in relation to PA type (i.e., team sports, individual sports). We found that, out of the total participant group, 81.4% participated in high-intensity team PA, while 93.9% participated in high-intensity individual PA. This indicates a general preference for individual sports over team sports, which is consistent with previous research [14,19]. However, team sports are more supportive in terms of the strategy of supporting PA and fulfilling PA recommendations, as it represents a factor that increases the probability of increasing the level of PA up to 2.5 times. This apparent discrepancy between higher preferences for individual activities and greater levels of PA support offered through team activities may indicate insufficient use of the potential of team sports and room for improvement in terms of the total amount of PA. However, the role of team sports, as well as the role of organized sports generally, is often overestimated, as pointed out by Badura et al. [59].

Hulteen et al. [50] reported that, to date, no global synthesis of individual PA preferences has been conducted. Devís-Devís, Beltrán-Carrillo, and Peiró-Velert [60] highlighted the interplay between personal and social factors, including preferences in adolescent (dis-)engagement in PA and sports. These factors appear to have an important impact on the construction of active identities (i.e., identity of active person) during adolescence.

Overall, there is significant potential to create a more complete picture of PA preferences at various life stages. This could provide policy-makers, exercise professionals, interventionists, and physical educators and coaches with useful information to aid PA promotion efforts, which, in turn, could increase current PA levels of any target group to improve health promotion strategies and social policies.

### Strengths and Limitations

The cross-sectional nature of the data, as well as the self-reported nature of data can be considered as major limitations of the present study. Other limitations are the usage of the Compendium of Physical Activities (with potential large errors in the approximation of PA intensity), the effect of seasonality (i.e., varying preferences in winter and summer months) and the time demands of the research study design.

On the other hand, the research design and combination of the implemented methods can be considered as the strength of the study.

## 5. Conclusions

Team sports appear to be a better strategy for meeting PA recommendations and should be promoted more within PA interventions, with the aim of improving lifestyle and quality of life. There was a significant association found between intensity level in team sports and PA per week. Involvement in high-intensity team sports increases the likelihood of being more physically active per week by 2.5 times. No significant association was found between intensity level in individual sports and PA per week.

There was only a small shift in the activity rankings throughout the surveyed age categories. Based on the literature review, the major differences in sports preferences can be visible considering the older age categories. The knowledge of micro-determinants of each type of activity (i.e., individual or team) can serve as a better moderator for more successful PA promotion strategies.

This manuscript is not attempting to prioritize particular activities over others, but rather it aims to point out the importance of considering the sport preferences factor as the moderator or PA promotion strategies and to uncover the potential of the precise aiming of these strategies.

## Figures and Tables

**Table 1:** Sports activities–division cut-off points.

Team Sports (METs)		Individual Sports (METs)	
Handball	12.0	Squash	12.0
Water polo	10.0	Martial arts	10.0
Dodgeball	8.0	Athletics	9.0
Soccer/football	8.0	Swimming	8.5
Hockey	8.0	Cycling	8.0
Floorball	7.5	Dance	6.5
Basketball	6.0	Tennis	6.0
Ultimate frisbee	5.0	Fitness activities	5.5
Footnet	4.0	Badminton	4.5
Volleyball	3.0	Golf	4.5

Note: cut-off points based on Ainsworth et al. [45,46].

**Table 2:** Distribution of physically active and inactive individuals.

			Czech Republic		Slovakia	
			*n*	%	*n*	%
	Total sample					
		Less active	318	74.8	319	72.3
		Active	108	25.2	122	27.7
	Gender					
	Boys	Less active	158	74.9	160	65.3
		Active	53	25.1	85	34.7
	Girls	Less active	160	74.4	159	81.1
		Active	55	25.6	37	18.9
	Grade level					
Boys	5th grade	Less active	92	84.6	85	85.5
		Active	17	15.4	14	14.5
	9th grade	Less active	94	92	118	81.7
		Active	8	8	28	18.3
Girls	5th grade	Less active	100	92.9	73	87.3
		Active	8	7.1	11	12.7
	9th grade	Less active	101	94.1	107	95.8
		Active	6	5.9	5	4.2

**Table 3:** Number of physically active days per week by country and gender.

		Czech Republic		Slovakia	
Number of Days Per Week		*n*	%	*n*	%
Boys	0	12	5.7	15	6.1
	1	13	6.2	11	4.5
	2	30	14.1	27	11.1
	3	39	18.5	49	20
	4	29	13.7	29	11.8
	5	35	16.6	29	11,8
	6	21	10	32	13.1
	7	32	15.2	53	21.6
Girls	0	17	7.9	12	6.1
	1	19	8.8	16	8.2
	2	32	14.9	34	17.3
	3	31	14.5	39	19.9
	4	39	18.1	32	16.3
	5	22	10.2	26	13.3
	6	14	6.5	11	5.6
	7	41	19.1	26	13.3

**Table 4:** Sample dichotomization considering activity type (team or individual sports).

	Total Sample (*n* = 867)	Czech Republic (*n* = 426)	Slovakia (*n* = 441)
Gender (boys): *n* (%)	456 (52.6)	211 (49.5)	245 (55.6)
Physical activity dich: *n* (%)			
active	85 (9.8)	32 (7.5)	53 (12.0)
Intensity in team sports dich: *n* (%)			
high intensity	706 (81.4)	325 (76.4)	378 (85.7)
Intensity in individual sports dich: *n* (%)			
high intensity	814 (93.9)	397 (93.1)	418 (94.7)

Note: Physical activity dichotomization (dich): active = ≥ 6 days activity per week, less active = < 6 days activity per week.

**Table 5:** Logistic regression model of physical activity level and activity type by intensity level.

	PA OR (95% CI)
Team sports	
low intensity (Ref)	
high intensity	2.48 (1.16–5.27) *
Individual sports	
low intensity (Ref)	
high intensity	2.61 (0.62–10.99) ^ns^

Note: *—statistical significance (*p* < 0.05); ns—not significant.

**Table 6:** The most preferred sports by grade level.

**11-Year-Olds—5th Graders**				
	**Boys**		**Girls**	
**Ranking**	**Czech Republic**	**Slovakia**	**Czech Republic**	**Slovakia**
1	Dodgeball	Football	Dodgeball	Dodgeball
2	Football	Floorball	Handball	Football
3	Floorball	Dodgeball	Football	Basketball
4	Handball	Basketball	Basketball	Handball
5	Basketball	Ice hockey	Volleyball	Volleyball
**15-Year-Olds—9th Graders**				
	**Boys**		**Girls**	
**Ranking**	**Czech Republic**	**Slovakia**	**Czech Republic**	**Slovakia**
1	Football	Football	Dodgeball	Dodgeball
2	Floorball	Floorball	Volleyball/Basketball	Volleyball
3	Dodgeball	Basketball	Football/Floorball	Football
4	Basketball	Volleyball	Handball	Basketball
5	Volleyball	Dodgeball	Ultimate frisbee	Handball

## Data Availability

No new data were created or analyzed in this study. Data sharing is not applicable to this article.
